# Nephrological Complications in Hemoglobinopathies: SITE Good Practice

**DOI:** 10.3390/jcm12237476

**Published:** 2023-12-02

**Authors:** Giovan Battista Ruffo, Rodolfo Russo, Tommaso Casini, Letizia Lombardini, Valeria Orecchia, Vincenzo Voi, Raffaella Origa, Gian Luca Forni, Monia Marchetti, Antonia Gigante, Giacomo Garibotto, Aurelio Maggio, Lucia De Franceschi

**Affiliations:** 1 U.O. Ematologia con Talassemia, ARNAS Civico Di Cristina Benfratelli, 90127 Palermo, Italy; 2 IRCSS AOU San Martino, Università degli Studi di Genova, 16146 Genova, Italy; rodolfo.russo@hsanmartino.it; 3 Centro Talassemie Ed Emoglobinopatie, Ospedale “Meyer”, 50139 Firenze, Italy; tommaso.casini@meyer.it; 4 Centro Nazionale Trapianti, Istituto Superiore di Sanità, 00161 Roma, Italy; letizia.lombardini@iss.it; 5 Clinica Pediatrica, Thalassemia e Malattie Rare, “Microcitemico A. Cao”, 09121 Cagliari, Italy; valeria.orecchia@aob.it; 6 Dipartimento di Scienze Cliniche e Biologiche, Centro delle Emoglobinopatie, Università di Torino, Ospedale San Luigi Gonzaga, 10125 Torino, Italy; vincenzo.voi@unito.it; 7 Talassemia, Ospedale Pediatrico Microcitemico ‘A. Cao’, ASL8, Università di Cagliari, 09121 Cagliari, Italy; raffaella.origa@unica.it; 8 Ematologia Centro della Microcitemia e delle Anemie Congenite—E.O. Ospedali Galliera, 16128 Genova, Italy; gianlucaforni14@gmail.com; 9 Hematology Unit, Azienda Ospedaliera Ss Antonio e Biagio e Cesare Arrigo, 15121 Alessandria, Italy; moniamarchettitamellini@gmail.com; 10 Società Italiana Talassemie d Emoglobinopatie (SITE), Fondazione per la Ricerca sulle Anemie ed Emoglobinopatie in Italia—For Anemia, 16124 Genoa, Italy; antonia.gigante@foranemia.org; 11 Dipartimento di Medicina Interna, Università degli Studi di Genova, 16132 Genova, Italy; gari@unige.it; 12 Unità Operativa Complessa Ematologia per le Malattie Rare del Sangue e degli Organi Ematopoietici, Azienda Ospedaliera Ospedali Riuniti Villa Sofia-V., 90146 Palermo, Italy; md.amaggio@gmail.com; 13 Dipartimento di Medicina, Università degli Studi di Verona-Azienda Ospedeliera Verona, 37124 Verona, Italy; lucia.defranceschi@univr.it

**Keywords:** hedging, transaction costs, dynamic programming, risk management, post-decision state variable

## Abstract

Background. Hemoglobinopathies, among which thalassemic syndromes (transfusion-dependent and non-transfusion dependent thalassemias) and sickle cell disease (SCD), are the most widespread monogenic diseases worldwide. Hemoglobinopathies are endemic and spread-out all-over Italy, as result of internal and external migration flows. Nowadays, the increase therapeutic options associated to the general aging of patients with hemoglobinopathies related to the improvement in clinical management, contribute to the abnormalities in kidney function going from blood and urine test alterations to chronic kidney disease and end stage renal disease. Methods. Here, we carried out a revision of the literature as panel of recognized experts in hemoglobinopathies with the consultancy and the revision of two nephrologists on kidney alteration and kidney disease in patients with TDT, NTDT and SCD. This is part of the action of the Italian society for the study of thalassemia and hemoglobinopties (SITE). The purpose of this “good practice (GP)” is to provide recommendations for follow-up and therapy for the management of kidney alterations in patients with TDT, NTDT and SCD. The literature review covers the period 1.1.2016 to 31.12.2022. In consideration of the rarity of these diseases, the analysis was extended from 5 to 7 years. Moreover, in the absence of relevant scientific papers in the identified time frame, we referred to pivotal or population studies, when available. Finally, in the absence of evidence-based data from prospective and randomized trials, the authors had to refer to expert opinion (expert consensus) for many topics. Results. We generated question and answer boxes to offer a friendly consultation, using color code strategy and focused answers. Conclusions. The present GP will help in improving the clinical management, and the quality of care of patients with hemoglobinopathies.

## 1. Introduction

Hemoglobinopathies, among which are thalassemia syndromes and sickle cell disease (SCD), are the most widespread monogenic diseases worldwide [1,2,3]. Italy is an endemic country for these pathologies, which are now present throughout the national territory as a result of internal and external migration flows from other endemic areas such as Sub-Saharan Africa, Central and South America, and South-East Asia [1,4].

The introduction of different medical treatment strategies such as oral iron chelating agents in thalassemia, screening for hepatitis C in blood transfusion units, and specific antiviral therapy, as well as the wide use of hydroxyurea in drepanocytosis associated with screening for vascular cerebropathy and antibiotic prophylaxis at a pediatric age, improved the average survival in adults and children [5,6,7,8], bringing out new organ complications. In addition to hepatocarcinoma [9], renal complications are a new frontier of action, both for their early identification and for follow-up and treatment.

Alterations of renal function are described early in SCD subjects, presenting with age-dependent non-selective proteinuria, reported in approximately 50% of patients between 36 and 45 years [10,11,12]. The evolution in forms of terminal renal insufficiency characterizes about 4.2% of the subjects affected by SCD in the homozygous form, SS [10,11,12]. Furthermore, renal function more rapidly declines in patients with SCD than in those with the sickle cell trait [13,14].

Renal damage is caused by the occlusion of glomerular and peritubular capillaries during a sickle cell crisis, resulting in the loss of nephrons with initial hypertrophy of the surviving glomeruli, tubulo-interstitial alterations of the ischemic type with functional impairment (defect of the urinary concentration process, metabolic acidosis, and hyperkalemia), and an increase in proteinuria up to the nephrotic value (proteinuria > 3.5 g/24 h). Other observed histological pictures are membrano-proliferative glomerulonephritis and thrombotic microangiopathy. Variants of APOL1, HMOX1, HBA1, and HBA2 have a role in the onset and progression of SCD-associated nephropathy ([13]; details on polymorphisms and mutations are reported in the cited review).

In subjects affected by thalassemia, the alterations of renal function could be attributable to the hemodynamic effects and hypoxic tubular damage induced by anemia [15,16] and iron overload [17,18,19]. The other possible cause is determined by iron chelators (deferoxamine and deferasirox), which, in rare cases, may be responsible for toxic or immune-mediated tubular damage [20,21]. In most cases of the alteration of renal function associated with the use of deferasirox, deterioration in the glomerular filtration rate was observed to be mild, reversible, and non-progressive [22]. From a pathophysiologic point of view, a renal hemodynamic alteration can be hypothesized, which was attributed to iron “relative depletion” with the activation of the tubuloglomerular feedback [23,24].

In conclusion, renal disease is a possible complication of thalassemia, considered as both transfusion-dependent (TDT) and non-transfusion-dependent (NTDT) thalassemias, and sickle cell disease. In the present document, we consider HbH disease as part of the NTDT group [25,26]. The continuous improvement of general clinical management increases the survival of this population but, paradoxically, makes the appearance of renal alterations more frequent due to the aging process, the accumulation of comorbidities, and the complexity of the drug therapy [27,28]. The final outcome of the renal complication is a worsening of the general prognosis of the patients, with an increase in morbidity and mortality, especially for cardiovascular causes.

The purpose of this “good practice” article is to briefly describe the various aspects of kidney disease in hemoglobinopathies and to provide recommendations for follow-up and therapy for the management of patients with TDT, NTDT, and SCD.

The literature review covers the period from 1.1.2016 to 31.12.2022; in consideration of the rarity of the pathologies, it was extended from 5 to 7 years. Moreover, in the absence of relevant scientific papers from the identified time frame, we referred to pivotal or population studies, when available. Finally, in the absence of evidence-based data from prospective and randomized trials, the panel of authors had to refer to expert opinion (expert consensus) for many topics [29].

The grading used in the present good practice is as follows:

- grade IA: strong recommendation based on strong evidence certainties and meta-analyses;
- grade IB: strong recommendation based on strong evidence certainties;
- grade IIA: strong recommendation based on moderate evidence certainties;
- grade IIB: strong recommendation based on moderate–weak evidence certainties;
- grade IIC: strong recommendation based on weak evidence certainties;
- grade IIIA: conditional recommendation based on strong evidence certainties;
- grade IIIB: conditional recommendation based on moderate evidence certainties;
- grade IIIC: conditional recommendation based on weak evidence certainties;
- grade IV: conditional recommendation based on expert indication.

## 2. Methods

In consideration of the rarity of hemoglobinopathies, SITE undertook a project aimed at integrating, with a systematic method, the lack of scientific papers to reach an adequate degree of consensus on recommendations for the clinical “management” of renal complications in this patient setting.

The Management Committee of SITE selected and brought together a multidisciplinary and multiprofessional group made up of experts in hemoglobinopathies and experts in nephropathies and organ transplantation, flanked by experts with methodological and organizational expertise, in order to create recommendations based on the integration of available scientific evidence together with expert opinion, with the aim of supporting clinicians in the decision-making process, thus improving the appropriateness of the therapies.

The members making up the scientific panel come from the following areas of expertise: 5 clinicians, who are experts on hemoglobinopathies; 1 nephropathy specialist expert in the management of patients with hemoglobinopathies; and 1 specialist expert in transplants belonging to the National Transplant Center.

In 2021, the Management Committee of SITE identified the members of the multidisciplinary panel, entrusting the coordination of the panel to two experts in hemoglobinopathies.

The panel of experts first divided the clinical problem into specific areas of interest and, for each area, identified specific scenarios and formulated the corresponding clinical questions (Supplementary Material File S1). The panel chose to refer the questions to the population with hemoglobinopathies in case the mechanism or characteristics of the object of the question were the same for thalassemia and sickle cell disease (SCD). Whenever the mechanism or characteristics of the object of the question were different for thalassemia and SCD, the panel differentiated the question for the two specific populations.

In order to increase the readability of the document, the panel labeled the study populations with three different color codes: gray for hemoglobinopathies, blue for thalassemia, and green for SCD.

The available evidence-based guidelines published between 1 January 2016 and 31 December 2022 were identified, evaluated, and selected; in particular, the following guidelines/good practice were selected:

- S.I.T.O. guidelines (https://www.societaitalianatrapiantidiorgano.com/linee-guida/) (accessed on 25 October 2022);
- KDIGO guidelines (https://kdigo.org/guidelines/) (accessed on 12 Febrary 2023;
- SITE good practice (https://manage.site-italia.org/scienza-e-formazione/buone-pratiche-site.html) (accessed on 2 July 2022)

More specific research, published between 1 January 2016 and 31 December 2022, related to nephrological complications in hemoglobinopathies was added to this.

The research was carried out through a systematic review of four main data sources: specific databases of guidelines; international health agencies producing guidelines; bibliographic databases only referring to guidelines; generalist databases (Scottish Intercollegiate Guidelines Network (SIGN), National Institute for Clinical Excellence (NICE), Pubmed, and Cochrane); the keywords used (both as MeSH terms and as non-MeSH terms) for the literature search are thalassemia, sickle, hemoglobinopathies, diagnosis, kidney, renal function, iron chelation–thalassemia, non-transfusion-dependent thalassemia, kidney transplantation, dialysis, cystatin, and deferasirox.

The search was completed manually and by questioning the panel experts about any “missing papers”.

A critical evaluation of evidence in terms of quality, up-to-dateness, and debated topics was carried out by the panel, who deemed it necessary to integrate what was selected from clinical studies focused on specific evidence for hemoglobinopathies.

**Figure 1:**
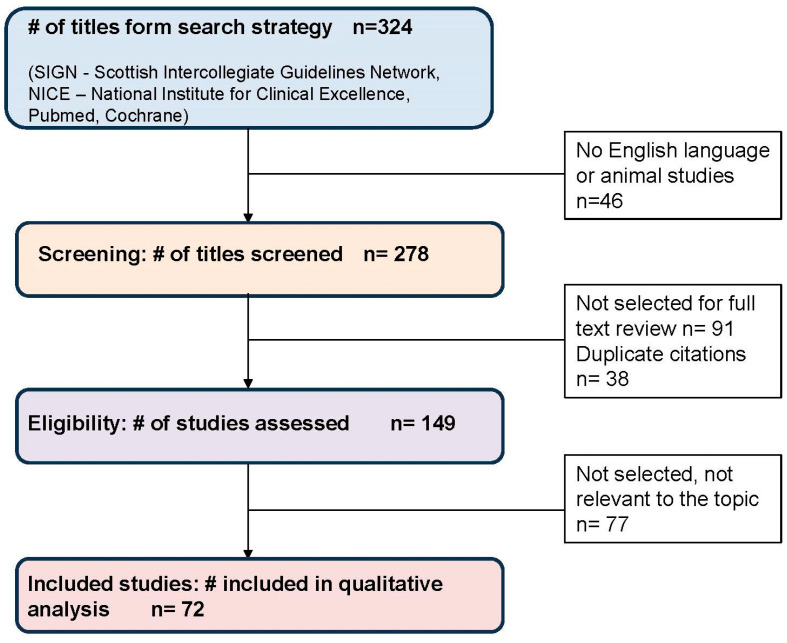
PRISMA flow-diagram for screening process of the literature. #: number; PRISMA: Preferred Reporting Items for Systematic Reviews and Meta-Analyses.

The literature search identified a total of 149 titles and abstracts. After a first evaluation—carried out based on abstracts—and a second one—carried out based on full texts—a total of 149 reference documents were considered to be relevant (Figure 1).

After completing the literature review evaluation, the authors answered the questions, specifying the evidence used to support the answer.

The authors presented and discussed what was prepared during eight plenary meetings held virtually between March 2021 and December 2022.

Following the presentation of the answers, an informal process took place to reach consensus on the strength and direction of the recommendations.

### Grading Scheme

For the evaluation of the evidence relating to clinical efficacy, the Evidence-Based Medicine (EBM) approach, which establishes a linear link between the quality of the evidence and the strength of the recommendation, was used and adopted for this purpose, dividing evidence based on the study design evaluation and on evidence levels.

For a transparent and systematic evaluation of the literature, the panel considered it appropriate to use a set of instruments to support the decision-making process in relation to the formulated clinical questions (Supplementary Material File S1), as outlined by the Grading of Recommendations Assessment, Development and Evaluation Evidence to Decision (GRADE EtD) frameworks, according to the study of H.J. Schunemann et al.

Therefore, the evidence is divided into

- strong and meta-analyses—systematic reviews and meta-analyses;
- strong—clinical studies with a randomized control group (RTC);
- moderate—clinical studies with a control group;
- moderate weak—cohort studies, case control studies, and observational studies;
- weak—case reports, case studies, and expert opinion.

Evidence levels are divided into

A data derived from several systematic reviews and meta-analyses;
B data derived from only one systematic review and meta-analysis or from various clinical studies with a randomized control group (RTC);
C data derived from observational, retrospective clinical studies or expert opinion.

The following list shows the grading used by the panel in this Good Practice:

- grade IA: strong recommendation based on strong evidence certainties and meta-analyses;
- grade IB: strong recommendation based on strong evidence certainties;
- grade IIA: strong recommendation based on moderate evidence certainties;
- grade IIB: strong recommendation based on moderate–weak evidence certainties;
- grade IIC: strong recommendation based on weak evidence certainties;
- grade IIIA: conditional recommendation based on strong evidence certainties;
- grade IIIB: conditional recommendation based on moderate evidence certainties;
- grade IIIC: conditional recommendation based on weak evidence certainties;
- grade IV: conditional recommendation based on expert indication.

The indications by the panel of authors were formulated based on common clinical experience, even in the absence of evidence or in the absence of sufficient supporting evidence, on issues deemed relevant to clinical practice.

The recommendations were written in clear and unambiguous language. Where necessary, notes were added including information on limitations and conditions of applicability, as well as details on target populations, interventions, settings, and outcomes.

In order to improve wording, solve ambiguities, remove futile or potentially dangerous statements, and suggest comments and criticalities, an editing process was performed.

The final version of the document was forwarded, for external review, to independent experts and representatives of patient associations in order to receive their comments and proposals for modification or supplementation.

The comments received by revisers were considered by the authors’ panel, who replied to comments and decided which changes should be made to the text based on such comments.

GP guidelines shall be updated every three years, starting from the date of publication. The methodology followed in the update will be the same as used in the present version.

The literature search shall begin from the date the present search was carried out.

Once the GP document is deemed suitable for publication, it will be published on the SITE website; it will also be presented at the main conferences on hemoglobinopathies and will be translated and submitted for publication to an international peer-reviewed journal.

## Data Availability

Analysis of the literature are available under request to www.site-italia.org.

## Supplementary Materials

The following supporting information can be downloaded at: https://www.mdpi.com/article/10.3390/jcm12237476/s1, File S1: clinical questions. Figure S1: Flow-chart for clinical management of albuminuria in patients with hemoglobinopathies. Figure S2: Flow-chart for clinical management of iron chelation therapy in patients with hemoglobinopathies and enal function alteration.

## Abbreviations

TDT	transfusion dependent thalassemia
NTDT	non-transfusion-dependent thalassemias
SCD	sickle cell disease
EEX	erythroexchange
UACR	urinary albumin/creatinine ratio
UPCR	urinary protein/creatinine ratio
ANA	anti-nuclear antibody
ENA-Ab	extractable nuclear antigen antibody
RF	rheumathoid factor
PCR	protein/creatinine ratio
ACR	albumin/creatinine ratio
ESKD	end-stage kidney disease
eGFR	estimated glomerular filtration rate
ACEi	angiotensin-converting enzyme inhibitor
MRA	mineral corticoid antagonist
SGLT2	sodium–glucose cotransporter 2 inhibitor
NSAIDs	nonsteroidal anti-inflammatory drugs
EMA	European Medicines Agency
SITE	Società Italiana Talassemie ed Emoglobinopatie
SITO	Società Italiana Trapianti d’Organo
ARNI	angiotensin receptor–neprilysin inhibitors
ARBs	angiotensin receptor blockers
ANCAs	antineutrophil cytoplasmatic antibodies
C3-C4	complement fraction 3 and fraction 4
