# Supplementary material for: Nephrological Complications in Hemoglobinopathies: SITE Good Practice
Source: J Clin Med. 2023 Dec 2;12(23):7476. doi: 10.3390/jcm12237476 (PMC10707125; doi:10.3390/jcm12237476)

Figure S1. Flow-chart for clinical management of albuminuria in patients with hemoglobinopathies

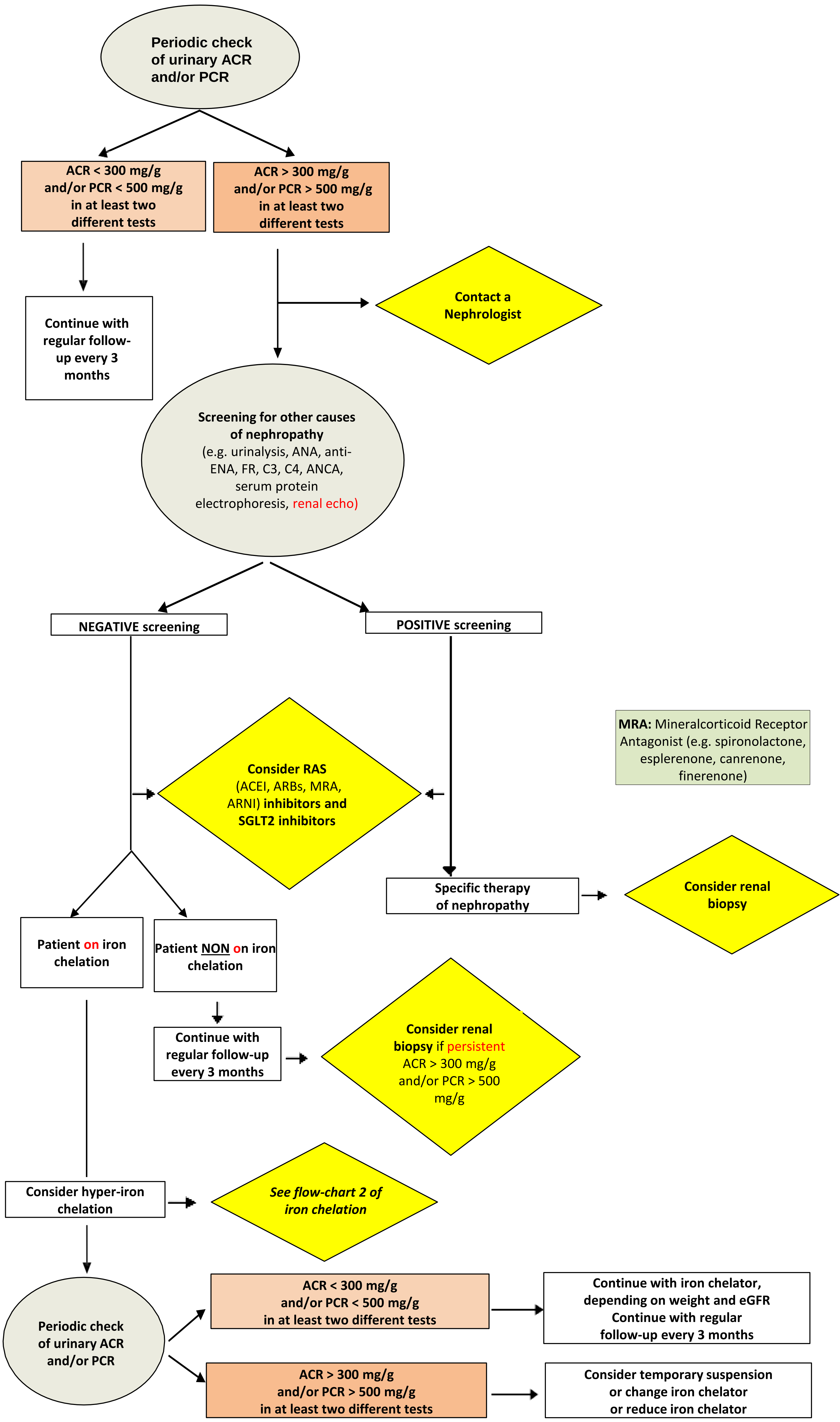

Figure S2. Flow-chart for clinical management of iron chelation therapy in patients with hemoglobinopathies and enal function alteration

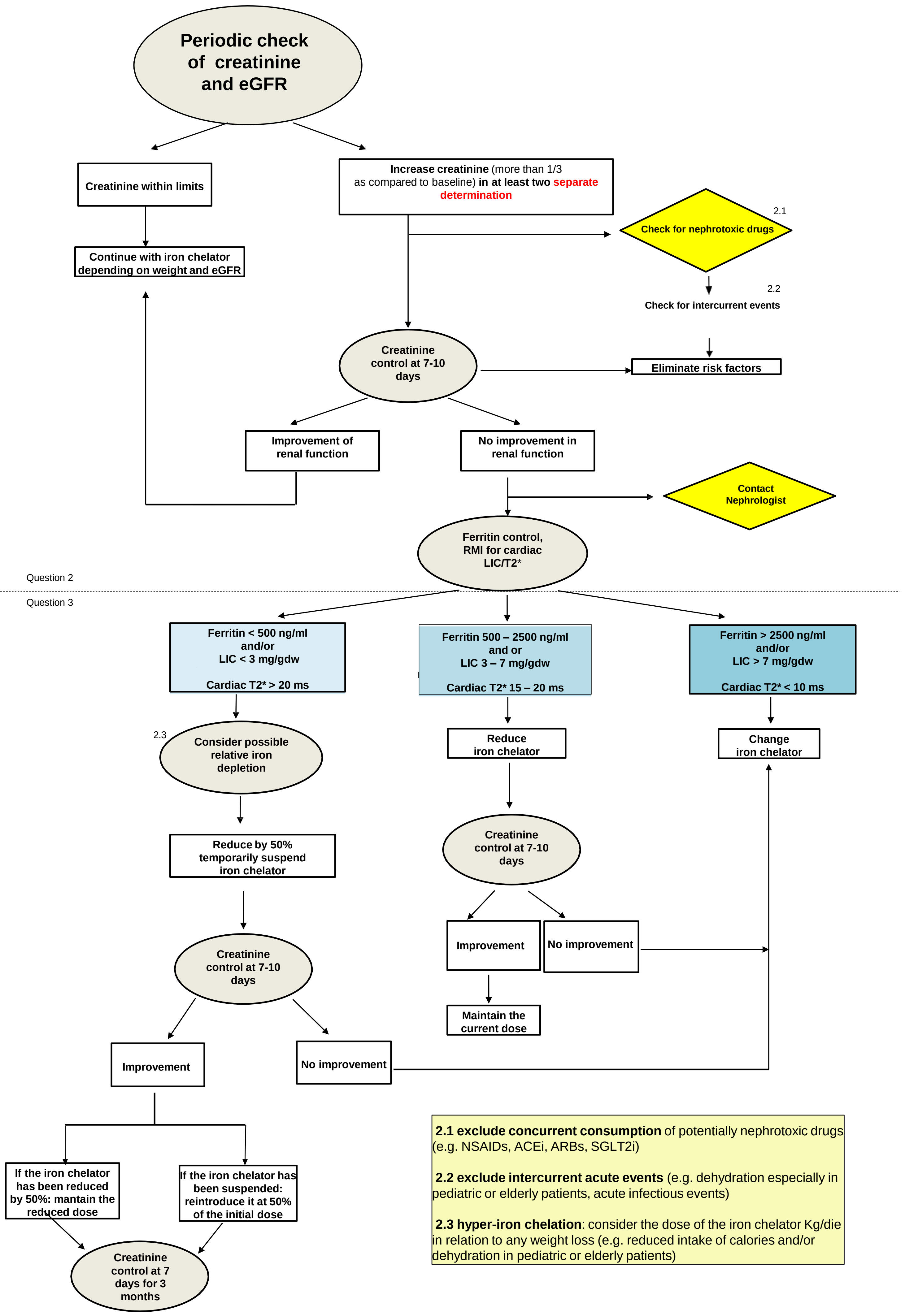

Supplement: Supplementary file 1 [file jcm-12-07476-s001.zip › jcm-2588230-Supplementary figures.pdf]
