# Supplementary material for: Nephrological Complications in Hemoglobinopathies: SITE Good Practice
Source: J Clin Med. 2023 Dec 2;12(23):7476. doi: 10.3390/jcm12237476 (PMC10707125; doi:10.3390/jcm12237476)
Supplement: Supplementary file 1 [file jcm-12-07476-s001.zip › jcm-2588230-Supplementary Material File S1.pdf]

Contacts

Società Italiana Talassemie ed Emoglobinopatie
e-mail: segreteriascientifica@site-italia.org – segreteriasite@ercongressi.it

Note for users

The present Good Pratice (GP) document is the tool through which the knowledges developed by biomedical research are transferred to daily clinical practice.
GP is based on the international standards of analysis to which they refer in a critical and contextualized manner: these standards must be able to express themselves, for each individual case, based on available clinical information, preferences expressed by patients and other contextual situations, accurately examined in light of the expertise of healthcare professionals. It is therefore up to the expertise and judgement of the professionals, who carefully listen to particular requests and consider the values expressed by patients, to establish which procedures or treatments are more appropriate to manage individual clinical cases.

Date of publication:
July 2023

Version No:
01

CLINICAL QUESTIONS

Question No 1 Which laboratory/instrumental tests are indicated to identify renal disease in a patient with hemoglobinopathy (e.g.: TDT, NTDT, SCD)?
Question No 1.1 How should patients with hemoglobinopathie be monitored?

Question No 2 How should patients with TDT/NTDT with worsening of kidney function be managed?

Question No 3 How should patients with SCD with worsening of kidney function be managed?

Question No 4 Which iron chelation therapy should be chosen, and which monitoring should be performed in a patient affected by chronic kidney disease with hemoglobinopathy (e.g.: TDT, NTDT, SCD)?

Question No 5 How should urinary tract infections be managed in patients with TDT/NTDT?

Question No 6 How should urinary tract infections b managed in patients with SCD?

Question No 7 How should renal calculosis be managed in patients with TDT/NTDT?

Question No 8 **How should renal calculosis be managed in patients with SCD?**

Question No 9 **How should renal transplant eligibility be managed in TDT/NTDT patients?**
Particular conditions - ***hyperimmunization***

Question No 10 **How should renal transplant eligibility be managed in SCD patients?**
Particular conditions - ***hyperimmunization***

Question No 11 **What measures should be adopted in TDT/NTDT patients in relation to the risk of rejection compared to the normal transplanted population?**

Question No 12 **What measures should be adopted in SCD patients in relation to the risk of rejection compared to the normal transplanted population?**

Question No 13 **Can TDT/NTDT patients be kidney donors?**

Question No 14 **Can SCD patients be kidney donors?**

Question No 15 **What measures should be adopted in the TDT/NTDT population in regard to the possible development of renal neoplasms?**

Question No 16 **What measures should be adopted in the SCD population in regard to the possible development of renal neoplasms?**

Question No 17 **When should Fanconi syndrome be considered in patients with hemoglobinopathy (e.g.: TDT, NTDT, SCD) and in iron chelation therapy?**

Question 1: Which laboratory/instrumental tests are indicated to identify renal disease in a patient with hemoglobinopathy (e.g.: TDT, NTDT, SCD)? (1-7)

Recommendation 1:
Type of grade: *grade IIA, strong recommendation based on moderate evidence certainties:*

In patients affected by hemoglobinopathy (e.g.: TDT, NTDT, SCD), the panel unanimously suggests to perform the following laboratory/instrumental tests on an annual basis for the early identification of nephropathy

- Creatinine, Cystatin C
- Glomerular filtrate with the CKD-EPI formula in adults and the Schwartz formula in children
- Venous EGA
- Standard urine test
- Urine culture in the presence of symptoms or urine alterations (hematuria and/or proteinuria, positivity for esterase and nitrites)
- ACR (albuminuria/creatinuria ratio) and PCR (proteinuria/creatinuria ratio) on spot urine (consider mg/gr as a unit of measure)
- Full abdomen ultrasound (consider doppler of renal arteries in case of creatinine alteration as in Figure S1-Flowchart 1)

Question No 1.1: How shoud patients with Hemoglobinopathies be monitored?

Recommendation 1.1:

Refer to Figure S1-**Flow-chart 1** for follow-up.

Special conditions:

- If the patient is on iron chelation with Deferasirox, refer to the data sheet for renal function checks during treatement (see also **Question 2**).

- If ACR > 300 mg/gr and/or PCR > 500 mg/gr in at least two different tests, consult the nephrologist and proceed as per **Flow-chart 1** for follow-up.

Question 2: How should patients with TDT/NTDT with worsening of kidney function be managed? (1, 8-16)

Type of grade: *grade IIA, strong recommendation based on moderate evidence certainties.*

There is no evidence in the literature related to an increased risk of end stage kidney disease (ESKD-End Stage Kidney Disease) in patients with TDT/NTDT ***(grade IIA, strong recommendation based on moderate evidence certainties)***.

An increased risk of chronic kidney disease is to be considered in TDT patients compared to NDTD patients depending on age, transfusion needs and current chelation therapy. Furthermore, age and chronic hemolysis are recognized as risk factors for renal damage in NTDT patients.

Recommendation 2:
For the clinical follow-up see **Question 1** and **Figure S1- Flow-chart 1.**

Question 3: How should SCD patients with worsening of kidney function be managed? (17-25)

Recommendation 3:
Type of grade: *grade IB, strong recommendatione based on strong evidence certainties.*

In the literature (retrospective, prospective and non-randomized observational) studies show an increased risk of ESKD in SCD subjects, both in childhood and in adulthood, with faster decline of eGFR in adults than in children

The presence of ESKD in SCD patients is a risk factor for premature death.

Patients with genotype SS and $Sb^{0/+}$ have a greater risk of ESKD than individuals with genotype SC or AS.

The following can be further risk factors:

- age (young adult)
- hemolysis (LDH)
- anemia - hypoxia (Hb)
- transfusion requirement
- hypertension
- proteinuria
- microhematuria

Patients with SCD and ESKD should be evaluated for renal transplantation.

Recommendation 3:
For the clinical follow-up see **Question 1** and **Figure S1- Flow-chart 1.**

Question 4: Which iron chelation therapy should be chosen, and which monitoring should be performed patient affected by chronic kidney disease with hemoglobinopathy (e.g.: TDT, NTDT, SCD)? (26-35)

Recommendation 4:
The panel unanimously suggests (see **Figure S2-Flow-chart 2**):

a) In case of Chronic Kidney Disease with GFR < 40 mL/min/1.73m2 choose between **Deferiprone Deferoxamine**

Type of grade: *grade IIC, strong recommendation based on weak evidence certainties*

b) In case of Chronic Kidney Disease with GFR ≥ 40 mL/min/1.73m2 choose from the three available iron chel (**Deferiprone**, **Deferasirox**, **Deferoxamine**) considering that there are no inferiority studies among the three iron chel in patients with hemoglobinopathy and chronic kidney disease with GFR ≥ 40 mL/min/1.73m2.

As far as **Deferasirox** is concerned, consider the increase in creatinine of at least one third from baseline in at leas different tests to be significant, then reduce the drug dose or try alternating drug regimens with another chelator (see **F chart 2**)*.

Type of grade: *grade IB, strong recommendation based on strong evidence certainties*

In particular, the panel unanimously suggests the following dosages:

o **Deferiprone** (75-100 mg/kg/die) is the only non-nephrotoxic chelator. It is not necessary to reduce the drug dose in advanced stages of kidney disease.
Maintain clinical – laboratory monitoring considering its narrow therapeutic window and possible adverse events agranulocytosis, arthralgias, gastrolesivity).

o **Deferoxamine** (20-50 mg/kg/die) is potentially nephrotoxic.
Maintain clinical – laboratory monitoring considering the possibility of an acute worsening of the dose-dependent function or in conjunction with serious infections.

o **Deferasirox** (12-14 mg/kg/die up to the dose tolerated by the patient, up to a maximum of 28 mg/kg/die) is poter nephrotoxic. If persistent signs of nephrotoxicity appear, refer to the drug's data sheet and to **Flow-chart 2**.
As far as Deferasirox is concerned, there are case reports in the literature describing its use in patients with ESKD a maximum dose of 25 mg/kg/die (deferasirox dispersible tablets) with no significant adverse events.
The panel reports that with eGFR clearance values < 60 mL/min/1.73m2 Deferasirox is offlabel according to indications; whereas its use is allowed with GFR ≥ 40 mL/min/1.73m2 according to FDA indications.
Rare cases of **Fanconi syndrome** and acute kidney damage have been reported, requiring the suspension of the (please refer to Question **10**).

Particular conditions:
- In SCD patients, iron-chelators can be combined with hydroxyurea treatment without increasing the risk of HU ind myelotoxicity.

Type of grade: *strong recommendation based on weak evidence certainties*.

- In diabetic TDT/NTDT patients with initail signs of nephropathy related to diabetes can be maintained on Deferas if it is already present as chronic thereapeutic treatment.

Type of grade: *strong recommendation based on weak evidence certainties.*

- Patients with possible Fanconi syndrome related to iron chelation treatment: stop the iron chelator (See also answ **question 10**).

Question 5: How should urinary tract infections be managed in patients with TDT/NTDT? (36)

Recommandation 5:

Type of grade: ***grade IV, conditional recommendation based on expert indication.***

In literature there is no evidence of an increased risk of urinary tract infections in patients with TDT/NTDT. Therefore the same risk factors of healthy population of the same age and gender must be considered (e.g. diabetes, urinary obstructive disease, calculosis, estroprogestinic therapy).

Question 6 **How should urinary tract infections be managed in patients with SCD?** (37, 38)

Recommendation 6:

Type of grade: *grade IIIC, conditional recommendation based on weak evidence certainties.*

In literature there is evidence of an increased risk of urinary tract infections in patients with SCD. It should be remembered that kidney is one of the target organs of SCD (e.g. impaired blood flow, papillary necrosis, reduced ability of the nephron to concentrate and acidify urine) and a higher prevalence of urinary tract infections is observed compared to the general population.

The panel unanimously suggests*:*
- If the patient is asymptomatic continue regular monitoring (see **Question 1** and **Figure S1- Flow-chart 1**), in addition it might be useful to carry out a urine dipstick test at each visit, and, in case of positivity for esterase/nitrites, a urine culture. Adequate hydration (1000-2000 ml/die) and regular bowel maintenance should always be recommended.
- If the patient is symptomatic (e.g. fever, sensory impairment/drowsiness particularly in children, general discomfort with no other explanation, pelvic discomfort, urinary symptoms), perform urine culture and undertake broad-spectrum antibiotic therapy only in case of fever (e.g. Amoxicillin/Clavulanic Acid); then proceed with targeted antibiotic therapy after identifying the pathogen through culture tests performed before starting broad-spectrum antibiotic therapy. Adequate hydration (1000-2000 ml/die) and regular bowel maintenance should always be recommended.

Imaging (CT, MRI, ultrasound) is indicated in patients with suspected acute or relapsing pyelonephritis to highlight the presence of renal calculosis or abscess lesions as in the normal population.

In the literature, the most frequently identified pathogens are: *Klebsiella spp, Staphylococcus aureus, Streptococci, Enterococcus spp, Pseudomonas, Proteus mirabilis, and Escherichia coli.*

Question 7: How should renal calculosis be managed in patients with TDT/NTDT? (3, 9, 26, 39-42)

Recommendation 7:
Type of grade: *grade IIB, strong recommendation based on moderate-weak evidence certainties*
In the literature there are only observational studies that show an increased risk of nefrolithiasis in TDT/NTDT patients compared to the general population, regardless of the use of iron chelators.
In case of renal calculosis the panel unanimously suggests:

· Metabolic study of calculosis in cooperation with the Nephrologist, according to the indications contained in "*Recommendations for the management of metabolic bone diseases in hemoglobinopathies*" by SITE (www.site-italia.org).

Moreover:

- If the patient is asymptomatic, ultrasound checkup on an annual basis;
- If the patient is symptomatic, consult the Nephrologist/Urologist and re-evaluate current therapy (e.g. iron chelator in case of iron overload (see **also Figure S2- Flow-chart 2 and Question 2);** thiazide diuretic in case of hypercalciuria; potassium citrate in case of hypocitraturia or uric acid stones; allopurinol or febuxostat in case of hyperuricemia associated with uric acid stones).
-

It should be remembered that in TDT the main risk factors are chronic hemolysis and chelation therapy, whereas in NTDT forms the main risk factor is splenectomy.
Stones are mainly made up of calcium oxalate and/or uric acid. The observation of a more frequent finding of hypercalciuria in TDT patients compared to NTDT patients is interesting.

Question 8: How should renal calculosis be managed in patients with SCD? (33, 43, 44)

Recommendation 8:

Type of grade: *grade IV, conditional recommendation based on expert indication.*

In the literature there is no evidence of an increase in nephrolithiasis in patiens affected by SCD, even though the tubular damage can be an independent damage factor.

Therefore, the same risk factors of healthy population of the same age and gender must be considered; the hyperuricemic effect of chronic treatment with hydroxyurea is the only peculiarity of SCD patients.

Question 9 **How should renal transplant eligibility be managed in TDT/NTDT patients?** (45-46)

Recommendation 9:

Type of grade: *grade IV, conditional recommendation based on expert indication.*

TDT/NTDT can be eligibile for kidney transplantation**.** The indication for transplantation is the same as for other types of patients with end-stage kidney disease. Please refer to the guidelines "*KDIGO Clinical Practice Guideline on the Evaluation and Management of Candidates for Kidney Transplantation*" (https://www.trapianti.salute.gov.it/imgs/C_17_cntPubblicazioni_15_allegato.pdf).

Particular conditions - *hyperimmunization*
Polytransfused patients with hemoglobinopathy are at high risk of *hyperimmunization**, in a very similar way to non-hemoglobinopathic subjects exposed to chronic transfusions or refractory to tretment with EPO or after rejection of kidney transplant. The hyperimmunization state can reduce the possibilities to find a donor match for the patient. Therefore, in the light of current knowledge and the scarcity of scientific data, each case should be evaluated individually by the multidisciplinary team involved in the inclusion of patients in the solid organ transplant waiting list.

**Hyperimmunization*: multiple transfusions can favour the development of auto/allo antibodies potentially limiting the identification of the donor and can expose the recipient to an increased risk of rejection. In case of hyperimmunization the patient can be included in the *National Hyperimmune Program 3.0 (PNI 3.0)* if he/she meets the specific requirements set forth in the protocol itself (https://www.trapianti.salute.gov.it/imgs/C_17_cntPubblicazioni_15_allegato.pdf).

I

Question 10. How should renal transplant eligibility be managed in SCD patients? (47-50)

Recommendation 10:

Tpe of grade: *grade IIC, strong recommendation based on weak evidence certainties.*

Patients with SCD and ESKD should be evaluated for kidney transplantation.

The panel unanimously suggests:
- Start/maintain manual or automated EEX regimens when including the patient in the transplant list (at least 31 months prior to transplantation) to obtain a percentage of HbS lower than 20-30% in order to prevent acute sickle-related complications that could compromise his/her permanence in the transplant list.
- Maintain hydroxyurea therapy until transplantation if used prior to transplantation. In relation to this it should be noted that there are no radomized studies concerning the use of hydroxyurea in this context.

Particular conditions - *hyperimmunization*
Polytransfused patients with hemoglobinopathy are at high risk of *hyperimmunization**, in a very similar way to non-hemoglobinopathic subjects exposed to chronic transfusions or refractory to treatment with EPO or after rejection of kidney transplant. The hyperimmunization state can reduce the possibilities to find a donor match for the patient. Therefore, in the light of current knowledge and the scarcity of scientific data, each case should be evaluated individually by the multidisciplinary team involved in the inclusion of patients in the solid organ transplant waiting list.

**Hyperimmunization*: multiple transfusions can favour the development of auto/allo antibodies potentially limiting the identification of the donor and can expose the recipient to an increased risk of rejection. In case of hyperimmunization the patient can be included in the *National Hyperimmune Program 3.0 (PNI 3.0)* if he/she meets the specific requirements set forth in the protocol itself (https://www.trapianti.salute.gov.it/imgs/C_17_cntPubblicazioni_15_allegato.pdf).

Question 11 What measures should be adopted in TDT/NTDT patients in relation to the risk of rejection compared to the normal transplanted population? (45, 46, 61, 52)

Recommendation 11:
Tpe of grade: *grade IV, conditional recommendation based on expert indication.*

In the literature there is no evidence of a higher risk of rejection for TDT and NTDT patients compared to the normal transplanted population.

Particular conditions - *hyperimmunization*
In consideration of the chronic transfusion regimen in TDT patients, the development of *hyperimmunization** is possible. This condition also occurs in the general non-thalassemic population who can become hyperimmunized (for example chronic transfusions in anemic, refractory patient or patient unsuitable for erythropoietin, previous kidney transplants). Hyperimmunization is a condition that might cause an increased risk of acute or chronic rejection.

There are no restrictions regarding the use of Thymoglobulins (ATG), as well as the newest anti-Interleukin-2 receptor monoclonal antibodies, in hyperimmunized TDT/NTDT patients. These strategies are introduced to reduce the risk of rejection of the transplanted organ, especially in patients with preexisting HLA antibodies at the time of surgery.

Therefore, in consideration of current knowledge and the scarcity of data in the literature, the panel unanimously suggests to follow the procedures aimed at increasing the survival of the transplanted organ envisaged for the general non-thalassemic population, with a joint discussion between the transplant team and the pathology expert.

**Hyperimmunization*: multiple transfusions can favour the development of auto/allo antibodies potentially limiting the identification of the donor and can expose the recipient to an increased risk of rejection. In case of hyperimmunization the patient can be included in the *National Hyperimmune Program 3.0 (PNI 3.0)* if he/she meets the specific requirements set forth in the protocol itself (https://www.trapianti.salute.gov.it/imgs/C_17_cntPubblicazioni_15_allegato.pdf).

Question 12 What measures should be adopted in SCD patients in relation to the risk of rejection compared to the normal transplanted population? (47, 49, 53-58)

Recommendation 12:
Type of grade: *grade IB, strong recommendation based on strong evidence certainties.*
SCD patients have a higher risk of rejection compared to general non-sickle population of the same age and gender**.**

The panel unanimously suggests:
- to maintain manual or automated EEX regimens for at least 48 months after the transplant. This approach reduces the risk of complications affecting the transplanted organ (e.g. delayed functional recovery, vaso-occlusive crises, rejection) and improves organ survival;
- to consider the risk of infection (e.g. CMV) and to implement prevention according to the procedure established for general non-sickle population undergoing renal transplantation (https://www.trapianti.salute.gov.it/imgs/C_17_cntPubblicazioni_15_allegato.pdf).

There are no randomized studies concerning the use of hydroyurea after kidney transplantation. However, there are data relating to the association of sirolimus and hydroxyurea in transplant patients with a positive effect on HbF levels, with no signs of myelotoxicity. Therefore, hydroxurea can be introduced during cycles of EEX starting from the last 3 months before the end of the transfusion procedures (at least 48 months post-transplant). In SCD patients, 48 months after transplantation, if acute sickle-related episodes occur, EEX procedures are indicated and should be used according to the algorithm for the management of acute events in patients with sickle cell disease (www.site-italia.org).

Particular conditions - *hyperimmunization*
In consideration of the chronic transfusion regimen in TDT patients, the development of *hyperimmunization** is possible. This condition also occurs in the general non-thalassemic population who can become hyperimmunized (for example chronic transfusions in anemic, refractory patient or patient unsuitable for erythropoietin, previous kidney transplants). Hyperimmunization is a condition that might cause an increased risk of acute or chronic rejection.
There are no restrictions regarding the use of Thymoglobulins (ATG), as well as the newest anti-Interleukin-2 receptor monoclonal antibodies, in hyperimmunized TDT/NTDT patients. These strategies are introduced to reduce the risk of rejection of the transplanted organ, especially in patients with preexisting HLA antibodies at the time of surgery.

Therefore, in consideration of current knowledge and the scarcity of data in the literature, the panel unanimously suggests to follow the procedures aimed at increasing the survival of the transplanted organ envisaged for the general non-thalassemic population, with a joint discussion between the transplant team and the pathology expert.

**Hyperimmunization*: multiple transfusions can favour the development of auto/allo antibodies potentially limiting the identificarion of the donor and expose the recipient to an increased risk of rejection. In case of hyperimmunization the patient can be included in the *National Hyperimmune Program 3.0 (PNI 3.0)* if he/she meets the specific requirements set forth in the protocol itself (https://www.trapianti.salute.gov.it/imgs/C_17_cntPubblicazioni_15_allegato.pdf).

Question 13 Can TDT/NTDT patients be kidney donors? (51, 52, 59)

Recommendation 13:
Type of grade: *grade IIC, strong recommendation based on weak evidence certainties.*

The panel unanimously suggests:

- in patients with NTDT and eGFR > 80 ml/min/1,73 m2 possible eligibility as donors for kidney transplant, once the transfusion history and any current or previous iron chelation therapy have been evaluated.
  In case this information is not available, the panel unanimously suggests to perform a kidney biopsy according to *S.I.T.O. guidelines* if risk factors for nephropathy of the general population are present (age > 60 years and comorbidities).

- In patients with TDT/NTDT and eGFR 60-79 ml/min/1,73 m2 evaluate the eligibility as donors for kidney transplant based on donor history and characteristics (e.g. young age, ethnicity, family history of kidney disease) according to *S.I.T.O. guidelines.*

- In patients with TDT/NTDT and eGFR < 60 ml/min/1,73 m2 exclude eligibility as donors for kidney transplant.

Therefore, given the limited availability of studies in the literature, the panel unanimously suggests a case-by-case evaluation by the kidney transplant team with the involvement of the expert in hematological pathology.

Question 14: Can patients with SCD be kidney donors? (60-63)

Recomendation 14:
Type of grade: *grade IIIB, conditional recommendation based on moderate evidence certainties:*

Living people with SCD cannot be accepted as kidney donors, regardless of their renal function.
Patients with sickle cell trait with normal renal function and urinalysis and albuminuria <30 mg/die should be evaluated as possible living donors.

The panel unanimously suggests*:*

- in patients with SCD and GFR > 80 ml/min/1,73 m2 possible eligibility as donors for cadaveric kidney transplant, once the transfusion history and any current or previous iron chelation therapy have been evaluated.
  In case this information is not available, the panel unanimously suggests to perform a kidney biopsy according to *S.I.T.O. guidelines.*

- In patients with SCD and eGFR 60-79 ml/min/1,73 m2 evaluate eligibility as donors for cadaveric kidney transplant after performing a kidney biopsy according to *S.I.T.O. guidelines*, taking into account:
  - duration of the disease
  - frequency of VOCs
  - number of hospitalizations
  - hypertension
  - proteinuria
  - severity of anemia

- In patients with SCD and eGFR < 60 ml/min/1,73 m2 exclude eligibility as donors for kidney transplant.

Therefore, given the limited availability of studies in the literature, the panel unanimously suggests a case-by-case evaluation by the kidney transplant team with the involvement of the expert in hematological pathology.

Question 15: What measures should be adopted in the TDT/NTDT population in regard to the possible development of renal neoplasms? (64, 65)

Recommendation 15:

Type of grade: *grade IV, conditional recommendation based on expert indication.*

In the literature there is no evidence of an increased risk of renal neoplasms in TDT/NTDT patients compared to non-thalassemic population of the same age and gender.

Therefore, the same risk factors of healthy population of the same age and gender should be considered (e.g. family history). The panel unanimously suggests to keep the follow-up unchanged, see **Question 1** and **Figure S1- Flow-chart 1.**

Question 16: What measures shoud be adopted in the SCD population in regard to the possible development of renal neoplasms? (64, 66, 67)

Recommendation 16:

Type of grade: *grade IV, conditional recommendation based on expert indication.*

In the literature there is no evidence of an increased risk of renal neoplasms in SCD patients (with genotype SS, Sb or SC) compared to non-sickle population of the same age and gender.

Therefore, the same risk factors of healthy population of the same age and gender should be considered (e.g. family history). The panel unanimously suggests to keep the follow-up unchanged, see **Question 1** and **Flow-chart 1**.

The panel unanimously refers to good practice for sickle cell disease carriers (AS), keeping in mind that these patients have an icreased risk of renal neoplasms (www.site-italia.org).

Question 17: When should Fanconi syndrome be considered in patients with hemoglobinopathy (e.g.: TDT, NTDT, SCD) and in iron chelation therapy? (68-72)

Recommendation 17:

Type of grade: *grade IV, conditional recommendation based on expert indication.*

Cases of acute renal failure after taking high doses of deferoxamine and during treatment with deferasirox, referable to acute toxic-type tubular damage, have been described. Concerning deferasirox, the panel highlights that acute reanal failure has been mainly observed in in elderly or in patients with diseases other than TDT such, myelodisplastic syndromes, renal or hepatic insufficiency. Rare cases of acute interstitial nephritis, hypersensitivity reactions have also been reported.

Fanconi syndrome clinical manifestations are.

- renal function alteration,

-severe metabolic acidosis,

-hypokalemia, hypophosphoremia

-reduced uricemia values.

Fanconi syndrome may appear within an average period of 17.8 months (range 1–36 months) after the initiation of deferasirox and is generally reversible within an average time of 3 weeks (range: 3 days – 6 weeks) following discontinuation of the therapy.

It is most commonly observed in patients younger than 16 years of age and in the elderly (age ≥ 65 years). It can also appear clinically with cases of isolated metabolic acidosis, the clinical presentation as hypophosphatemic osteomalacia is also possible. It should also be noted that NTDT/TDT and SCD patients may experience heart failure related to iron overload cardiomyopathy, developing cardiorenal syndrome and consequent acute and/or chronic renal failure.

The panel suggests stopping the iron chelator and to treat acute renal failure in collaboration with the nephrologist. Iron chelators different from the molecule causing Fanconi syndrome might be reintroduce after the recovery of kidney function up to pro-fanconi kidney function values

GOOD PRACTICE REFERENCES

1. Mahmoud AA, Elian DM, Abd El Hady NM, Abdallah HM, Abdelsattar S, Khalil FO, Abd El Naby SA.Assessment of Subclinical Renal Glomerular and Tubular Dysfunction in Children with Beta Thalassemia Major.Children (Basel). 2021 Feb; 8(2): 100.

2. Abd El-Khalik SR, Sharaby RM, Nasif E, Hamza MB, Ibrahim RR. Netrin-1 and clusterin: Innovative potential diagnostic biomarkers for early renal damage in β-thalassemia major children. IUBMB Life 2021 May;73(5):800-810

3. Arman Bilir Ö, Kirkiz S, Fettah A, Ok Bozkaya İ, Kara A, Çakar N, Yaralı N. Renal function and the oxidative status among children with thalassemia major and healthy controls: A cross-sectional study. Transfus Apher Sci 2020 Aug;59(4):102746.

4. Olaniran KO, Allegretti AS, Zhao SH, Nigwekar SU, Kalim S. Kidney Function Decline among Black Patients with Sickle Cell Trait and Sickle Cell Disease: An Observational Cohort Study. J AmSocNephrol2020 Feb;31(2):393-404.

5. Nnaji UM, Ogoke CC, Okafor HU, Achigbu KI.Sickle Cell Nephropathy and Associated Factors among Asymptomatic Children with Sickle Cell Anaemia.Int J Pediatr. 2020 Aug 14;2020:1286432.

6. Jeffrey D Lebensburger , Inmaculada Aban , Brandi Pernell, Malgorzata Kasztan, Daniel I Feig, Lee M Hilliard, David J Askenazi. Hyperfiltration during early childhood precedes albuminuria in pediatric sickle cell nephropathy. Am J Hematol. 2019 Apr;94(4):417-423.

7. Saif A, Soliman N, Abdelhamid A. Doppler assessment of renal hemodynamic alterations in homozygous sickle cell disease and sickle Beta-thalassemia. Ultrason Imaging. 2015 Jul;37(3):258-64. Doi: 10.1177/0161734614553831

8. Milo G, Feige Gross Nevo R, Pazgal I, Gafter-Gvili A, Shpilberg O, Gafter U, Erman A, Stark P. GFR in Patients with β-Thalassemia Major. Clin J Am Soc Nephrol. 2015 Aug 7;10(8):1350-6. doi: 10.2215/CJN.12181214. Epub 2015 May 11. PMID: 25964308; PMCID: PMC4527037.

9. Quinn CT, Johnson VL, Kim HY, Trachtenberg F, Vogiatzi MG, Kwiatkowski JL, Neufeld EJ, Fung E, Oliveri N, Kirby M, Giardina PJ; Thalassemia Clinical Research Network. Renal dysfunction in patients with thalassaemia. Br J Haematol. 2011 Apr;153(1):111-7. doi: 10.1111/j.1365-2141.2010.08477.x. Epub 2011 Feb 21. PMID: 21332704; PMCID: PMC4250090.

10. Ali BA, Mahmoud AM. Frequency of glomerular dysfunction in children with Beta thalassaemia major. Sultan Qaboos Univ Med J. 2014 Feb;14(1):e88-94. doi: 10.12816/0003341. Epub 2014 Jan 27. PMID: 24516760; PMCID: PMC3916283.

11. Lai ME, Spiga A, Vacquer S, Carta MP, Corrias C, Ponticelli C. Renal function in patients with β-thalassaemia major: a long-term follow-up study. Nephrol Dial Transplant. 2012 Sep;27(9):3547-51. doi: 10.1093/ndt/gfs169. Epub 2012 Jun 13. PMID: 22695832.

12. Tanous, O., Azulay, Y., Halevy, R. et al. Renal function in β-thalassemia major patients treated with two different iron-chelation regimes. BMC Nephrol 22, 418 (2021). https://doi.org/10.1186/s12882-021-02630-5

13. Hashemieh M, Radfar M, Azarkeivan A, Hosseini Tabatabaei SMT, Nikbakht S, Yaseri M, Sheibani K. Renal Hemosiderosis among Iranian Transfusion Dependent β-Thalassemia Major Patients. Int J Hematol Oncol Stem Cell Res. 2017 Apr 1;11(2):133-138. PMID: 28875008; PMCID: PMC5575726.

14. Nafea OE, Zakaria M, Hassan T, El Gebaly SM, Salah HE. Subclinical nephrotoxicity in patients with beta-thalassemia: role of urinary kidney injury molecule. Drug Chem Toxicol. 2022 Jan;45(1):93-102. doi: 10.1080/01480545.2019.1660362. Epub 2019 Sep 5. PMID: 31905029.

15. Al-Khabori M, Bhandari S, Al-Rasadi K, Mevada S, Al-Dhuhli H, Al-Kemyani N, Daar S. Correlation of iron overload and glomerular filtration rate estimated by cystatin C in patients with β-thalassemia major. Hemoglobin. 2014;38(5):365-8. doi: 10.3109/03630269.2014.944314. Epub 2014 Jul 30. PMID: 25074643.

16. Ziyadeh FN, Musallam KM, Mallat NS, Mallat S, Jaber F, Mohamed AA, Koussa S, Taher AT. Glomerular hyperfiltration and proteinuria in transfusion-independent patients with β-thalassemia intermedia. Nephron Clin Pract. 2012;121(3-4):c136-43. doi: 10.1159/000339787. Epub 2012 Dec 8. PMID: 23235469.

17. Platt O, Brambilla D, Rosse W, et al. Mortality in sickle cell disease-life expectancy and risk factors of early death. N Engl J Med. 1994; 330:1639-1644.

18. Roy NB, Fortin PM, Bull KR, et al. Interventions for chronic kidney disease in people with sickle cell disease. Cochrane Database Syst Rev. 2017 Jul 3;7(7):CD012380. doi: 10.1002/14651858.

19. Powars DR, Elliott-Mills DD, Chan L, et al. Chronic renal failure in sickle cell disease: risk factors, clinical course, and mortality. Annals of Internal Medicine. 1991; 115:614–620.

20. Zahr RS, Yee ME, Weaver J, et al. Kidney biopsy findings in children with sickle cell disease: a Midwest Pediatric Nephrology Consortium study. Pediatr Nephrol. 2019 Aug;34(8):1435-1445

21. Alkhunaizi AM, Al-Khatti AA, Al-Mueilo SH, Amir A, Yousif B. End-stage renal disease in patients with sickle cell disease. Saudi J Kidney Dis Transpl. 2017 Jul-Aug;28(4):751-757

22. Saraf SL, Zhang X, Kanias T, et al. Haemoglobinuria is associated with chronic kidney disease and its progression in patients with sickle cell anaemia. Br J Haematol. 2014 Mar;164(5):729-39

23. Niss O, Lane A, Asnani MR, et al. Progression of albuminuria in patients with sickle cell anemia: a multicenter, longitudinal study. Blood Adv. 2020 Apr 14;4(7):1501-1511

24. Olaniran KO, Allegretti AS, Zhao SH, et al. Kidney Function Decline among Black Patients with Sickle Cell Trait and Sickle Cell Disease: An Observational Cohort Study. J Am Soc Nephrol. 2020 Feb;31(2):393-404.

25. Ataga KI, Saraf SL, Derebail VK. The nephropathy of sickle cell trait and sickle cell disease. Nat Rev Nephrol. 2022 Jun;18(6):361-377. doi: 10.1038/s41581-022-00540-9.

26. Demosthenous C, Vlachaki E, Apostolou C, Eleftheriou P, Kotsiafti A, Vetsiou E, Mandala E, Perifanis V, Sarafidis P. Beta-thalassemia: renal complications and mechanisms: a narrative review. Hematology. 2019 Dec;24(1):426-438

27. Badeli H, Baghersalimi A, Eslami S, Saadat F, Rad AH, Basavand R, Papkiadeh SR, Darbandi B, Kooti W, Peluso I. Early Kidney Damage Markers after Deferasirox Treatment in Patients with Thalassemia Major: A Case-Control Study. Oxid Med Cell Longev. 2019 Apr 21;2019:5461617.

28. Bird ST, Swain RS, Tian F, Okusanya OO, Waldron P, Khurana M, Durmowicz EL, Ma Y, Major JM, Gelperin K. Effects of deferasirox dose and decreasing serum ferritin concentrations on kidney function in paediatric patients: an analysis of clinical laboratory data from pooled clinical studies. Lancet Child Adolesc Health. 2019 Jan;3(1):15-22.

29. Pinto VM, Poggi M, Russo R, Giusti A, Forni GL, Management of the aging beta-thalassemia transfusion-dependent population – The Italian experience. Blood Rev. 2019 Nov;38:100594

30. Scoglio M, Cappellini MD, D'Angelo E, Bianchetti MG, Lava SAG, Agostoni C, Milani GP. Kidney Tubular Damage Secondary to Deferasirox: Systematic Literature Review. Children (Basel). 2021 Dec 1;8(12):1104

31. Fradette C, Pichette V, Sicard É, Stilman A, Jayashankar S, Tsang YC, Spino M, Tricta F. Effects of renal impairment on the pharmacokinetics of orally administered deferiprone. Br J Clin Pharmacol. 2016 Oct;82(4):994-1001.

32. Beydoun HG, Saliba AN, Taher AT. Deferasirox in thalassemia patients with end-stage renal disease. Am J Hematol. 2016 Oct;91(10):E456-7.

33. Origa R, Piga A, Tartaglione I, Della Corte G, Forni GL, Bruederle A, Castiglioni C, Han J. Renal safety under long-course deferasirox therapy in iron overloaded transfusion-dependent β-thalassemia and other anemias. Am J Hematol. 2018 Jul;93(7):E172-E175

34. Wong TE, Valle J, PaulukonisS.Concurrent use of hydroxyurea and deferasirox in Californians with sickle cell disease.Health Sci Rep. 2021;4:e323

35. Karimi M, Avazpour A, Haghpanah S, Toosi F, Badie A. Evaluation of Proteinuria in β-Thalassemia Major Patients With and Without Diabetes Mellitus Taking Deferasirox. J PediatrHematol Oncol. 2017 Jan;39(1):e11-e14.

36. Ponticelli C, Musallam KM, Cianciulli P, Cappellini MD. Renal complications in transfusion-dependent beta thalassaemia. Blood Rev. 2010 Nov;24(6):239-44. doi: 10.1016/j.blre.2010.08.004. Epub 2010 Sep 20. PMID: 20850917.

37. Musonda T, Zulu M, Samutela M, Kalonda A, Mantina H, Okuku P, Sinkala M, Nkhoma P. Leucocytosis and Asymptomatic Urinary Tract Infections in Sickle Cell Patients at a Tertiary Hospital in Zambia. Anemia. 2020 Jun 2;2020:3792728. doi: 10.1155/2020/3792728. PMID: 32566287; PMCID: PMC7290901.

38. Expert Panel on Urological Imaging; Andrew D Smith, Paul Nikolaidis, Gaurav Khatri, Suzanne T Chong, Alberto Diaz DeLeon , Dhakshinamoorthy Ganeshan, John L Gore, Rajan T Gupta, Richard Kwun, Andrej Lyshchik, Refky Nicola, Andrei S Purysko, Stephen J Savage, Myles T Taffel, Don C Yoo, Erin W Delaney, Mark E Lockhart. ACR Appropriateness Criteria® Acute Pyelonephritis: 2022 Update J Am Coll Radiol.2022 Nov;19(11S):S224-S239. Doi: 10.1016/j.jacr.2022.09.017.

39. Bhandari S, Galanello R. Renal aspects of thalassaemia a changing paradigm. Eur J Haematol. 2012 Sep;89(3):187-97. doi: 10.1111/j.1600-0609.2012.01819.x. Epub 2012 Jul 21. PMID: 22687050

40. Smolkin V, Halevy R, Levin C, Mines M, Sakran W, Ilia K, Koren A. Renal function in children with beta-thalassemia major and thalassemia intermedia. Pediatr Nephrol. 2008 Oct;23(10):1847-51. doi: 10.1007/s00467-008-0897-8. Epub 2008 Jun 25. PMID: 18581145.

41. Motta I, Mancarella M, Marcon A, Vicenzi M, Cappellini MD. Management of age-associated medical complications in patients with β-thalassemia. Expert Rev Hematol. 2020 Jan;13(1):85-94. doi: 10.1080/17474086.2020.1686354. Epub 2019 Nov 12. PMID: 31661637.

42. Aliberti L, Gagliardi I, Gamberini MR, Ziggiotto A, Verrienti M, Carnevale A, Bondanelli M, Zatelli MC, Ambrosio MR. Beta-thalassaemia major: Prevalence, risk factors and clinical consequences of hypercalciuria. Br J Haematol. 2022 Sep;198(5):903-911. doi: 10.1111/bjh.18345. Epub 2022 Jun 29. PMID: 35768889; PMCID: PMC9542302.

43. Patel N, Farooqi A, Callaghan M, Sethuraman U. Urinary Tract Infection in Febrile Children with Sickle Cell Disease Who Present to the Emergency Room with Fever. J Clin Med. 2020 May 19;9(5):1531. doi: 10.3390/jcm9051531. PMID: 32438767; PMCID: PMC7290819.

44. Wong P, Polkinghorne K, Kerr PG, Doery JC, Gillespie MT, Larmour I, Fuller PJ, Bowden DK, Milat F. Deferasirox at therapeutic doses is associated with dose-dependent hypercalciuria. Bone. 2016 Apr;85:55-8. doi: 10.1016/j.bone.2016.01.011. Epub 2016 Jan 21. PMID: 26802257.

45. ME McPherson Yee et al. “Class I and II HLA antibodies in pediatric patients with thalassemia major”. Transfusion, 2016 Apr;56(4):878-84

46. H Oweira et. al. “Risk Factors of Rejection in Renal Transplant Recipients: A Narrative Review”, J. Clin. Med. 2022, 11, 1392.

47. Willis JC, Awogbade M, Howard J, et al. Outcomes following kidney transplantation in patients with sickle cell disease: The impact of automated exchange. PLOS ONE https://doi.org/10.1371/journal.pone.0236998 August 13, 2020 blood transfusion

48. Leeaphorn N, Thongprayoon C, Vaitla P, et al. Outcomes of Kidney Transplant Recipients with Sickle Cell Disease: An Analysis of the 2000-2019 UNOS/OPTN Database. J Clin Med. 2021 Jul 11;10(14):3063

49. Oweira H, Ramouz A, Ghamarnejad O, et al. Risk Factors of Rejection in Renal Transplant Recipients: A Narrative Review. J Clin Med. 2022 Mar 3;11(5):1392

50. Al-Khatti AA, Alkhunaizi AM. Additive effect of sirolimus and hydroxycarbamide on fetal haemoglobin level in kidney transplant patients with sickle cell disease. Br J Haematol. 2019 Jun;185(5):959-961

51. Umasankar Mathuram Thiyagarajan et al.“Thymoglobulin and Its Use in Renal Transplantation: A Review”. Am J Nephrol 2013;37:586–601 doi: 10.1159/000351643

52. Bamoulid et al. Anti-thymocyte globulins in kidney transplantation: focus on; current indications and long-term immunological side effects”.Nephrol Dial Transplant (2017) 32: 1601–1608

53. Okafor UH, Aneke E. Outcome and challenges of kidney transplant in patients with sickle cell disease. J Transplant. 2013;2013:614610. doi: 10.1155/2013/614610. Epub 2013 Apr 11.

54. Leeaphorn N, Thongprayoon C, Vaitla P, et al. Outcomes of Kidney Transplant Recipients with Sickle Cell Disease: An Analysis of the 2000-2019 UNOS/OPTN Database. J Clin Med. 2021 Jul 11;10(14):3063.

55. Bae S, Johnson M, Massie AB, et al. Mortality and Access to Kidney Transplantation in Patients with Sickle Cell Disease-Associated Kidney Failure. Clin J Am Soc Nephrol. 2021 Mar 8;16(3):407-414

56. Al-Khatti AA, Alkhunaizi AM. Additive effect of sirolimus and hydroxycarbamide on fetal haemoglobin level in kidney transplant patients with sickle cell disease. Br J Haematol. 2019 Jun;185(5):959-961

57. Brennan DC, Lippmann BJ, Shenoy S, Lowell JA, Howard TK, Flye MW. Living unrelated renal transplantation for sickle cell nephropathy. Transplantation. 1995 Mar 15;59(5):794-5

58. Gérardin C, Moktefi A, Couchoud C, et al. Survival and specific outcome of sickle cell disease patients after renal transplantation. Br J Haematol. 2019 Dec;187(5):676-680

59. Dinh, N.H., Beaupha, S.M.C. Safety, clinical and laboratory characteristics of donors with thalassemia minor in living donor kidney transplant: a case series. BMC Nephrol 22, 397 (2021). https://doi.org/10.1186/s12882-021-02609-2

60. Rossidis A, Lim MA, Palmer M, et al. Kidney Transplantation From a Donor With Sickle Cell Disease. Am J Transplant. 2017 Feb;17(2):569-571

61. Philipponnet C, Aniort J, Garrouste C, Kemeny JL, Hadj-Abdelkader M, Heng AE. Kidney Transplantation With a Sickle Cell Disease Donor. Kidney Int Rep. 2020 Aug 8;5(10):1836-1838.

62. Sureshkumar KK, Chopra B. Impact of Donor Ethnicity on Long-Term Kidney Transplant Outcomes: Analysis by Kidney Donor Profile Index Categories. Exp Clin Transplant. 2020 Apr;18(2):144-148

63. Rehman Su, Al-Amoudi A, Kelta M, Awad A, Bridges K, Al-Ghamdi SM. Kidney transplant from sickle cell trait donor to sickle cell trait recipient. Exp Clin Transplant. 2007 Dec;5(2):698-700.

64. Elliott A, Bruner E. Renal Medullary Carcinoma. Arch Pathol Lab Med. 2019 Dec;143(12):1556-1561. eng. Epub 20190311. doi:10.5858/arpa.2017-0492-RS. Cited in: Pubmed; PMID 30855171.

65. Torti SV, Torti FM. Iron and cancer: more ore to be mined. Nat Rev Cancer. 2013 May;13(5):342-55. doi: 10.1038/nrc3495. Epub 2013 Apr 18. PMID: 23594855; PMCID: PMC4036554.

66. Alvarez OA. Renal Medullary Carcinoma: The Kidney Cancer That Affects Individuals With Sickle Cell Trait and Disease. Journal of Oncology Practice. 2017;13(7):424-425. doi:10.1200/jop.2017.023820. Cited in: Pubmed; PMID 28697316.

67. Alvarez O, Rodriguez MM, Jordan L, Sarnaik S. Renal medullary carcinoma and sickle cell trait: A systematic review. Pediatr Blood Cancer. 2015 Oct;62(10):1694-9. eng. Epub 20150605. doi:10.1002/pbc.25592. Cited in: Pubmed; PMID 26053587.

68. Fraser J, Brook R, He T, Lewis D. Deferasirox-induced liver injury and Fanconi syndrome in a beta-thalassemia major male. BMJ Case Rep. 2020 Jul 9;13(7):e234542. doi: 10.1136/bcr-2020-234542. PMID: 32646935; PMCID: PMC7351284.

69. Yui JC, Geara A, Sayani F. Deferasirox-associated Fanconi syndrome in adult patients with transfusional iron overload. Vox Sang. 2021 Aug;116(7):793-797. doi: 10.1111/vox.13064. Epub 2021 Feb 2. PMID: 33529394.

70. Wei HY, Yang CP, Cheng CH, Lo FS. Fanconi syndrome in a patient with β-thalassemia major after using deferasirox for 27 months. Transfusion. 2011 May;51(5):949-54. doi: 10.1111/j.1537-2995.2010.02939.x. Epub 2010 Nov 15. PMID: 21077910.

71. Chuang GT, Tsai IJ, Tsau YK, Lu MY. Transfusion-dependent thalassaemic patients with renal Fanconi syndrome due to deferasirox use. Nephrology (Carlton). 2015 Dec;20(12):931-5. doi: 10.1111/nep.12523. PMID: 26016559.

72. Rheault MN, Bechtel H, Neglia JP, Kashtan CE. Reversible Fanconi syndrome in a pediatric patient on deferasirox. Pediatr Blood Cancer. 2011 Apr;56(4):674-6.doi: 10.1002/pbc.22711. Epub 2010 Dec 6. PMID: 21298760.
